# Micronized Organic Magnesium Salts Enhance Opioid Analgesia in Rats

**DOI:** 10.1371/journal.pone.0161776

**Published:** 2016-10-28

**Authors:** Magdalena Bujalska-Zadrożny, Kamila Kulik, Michał Ordak, Małgorzata Sasinowska-Motyl, Emilia Gąsińska, Anna de Corde, Agnieszka Kowalczyk, Mariusz Sacharczuk, Marek Naruszewicz

**Affiliations:** 1 Department of Pharmacodynamics, Centre for Preclinical Research and Technology, Medical University of Warsaw, Warsaw, Poland; 2 Chair and Department of Psychiatry, Medical University of Warsaw, Warsaw, Poland; 3 Department of Cytogenetics, Institute of Genetics and Animal Breeding, Polish Academy of Sciences, Magdalenka, Poland; 4 Department of Pharmacognosy and Molecular Basis of Phytotherapy, Medical University of Warsaw, Warsaw, Poland; Xi'an Jiaotong University School of Medicine, CHINA

## Abstract

**Purpose:**

As previously reported, magnesium sulphate administered parenterally significantly increased an opioid antinociception in different kinds of pain. Since the typical form of magnesium salts are poorly and slowly absorbed from the gastrointestinal tract we examined whether their micronized form could increase opioids induced antinociception.

**Methods:**

In behavioural studies on rats morphine, tramadol and oxycodone together with magnesium (lactate dihydrate, hydroaspartate, chloride) in micronized (particles of size D_90_ < 50 μm) and conventional forms were used. Changes in pain thresholds were determined using mechanical stimuli. The intestinal absorption of two forms of magnesium lactate dihydrate (at the doses of 7.5 or 15 mg ions) in the porcine gut sac model were also compared.

**Results:**

Micronized form of magnesium lactate dihydrate or hydroaspartate but not chloride (15 mg of magnesium ions kg^-1^) enhanced the analgesic activity of orally administered opioids, significantly faster and more effective in comparison to the conventional form of magnesium salts (about 40% for oxycodone administered together with a micronized form of magnesium hydroaspartate). Moreover, in vitro studies of transport across porcine intestines of magnesium ions showed that magnesium salts administered in micronized form were absorbed from the intestines to a greater extent than the normal form of magnesium salts.

**Conclusions:**

The co-administration of micronized magnesium organic salts with opioids increased their synergetic analgesic effect. This may suggest an innovative approach to the treatment of pain in clinical practice.

## Introduction

The treatment of pain remains an unsatisfactorily resolved problem within medicine. The algorithm of pain treatment is based on the rule that the stronger the pain, the more effective and more strongly acting must be the analgesic. This schematic is also known by its common name, “Analgesic Ladder”, on which individual rungs are occupied by nonsteroidal analgesics, then weak opioids, and finally potent opioid analgesics [1]. This schematic also encompasses the use of adjuvants, which may enhance opioid activity. In the absence of other, equally effective analgesic drugs; opioids should be increasingly used in palliative care. Unfortunately, there would seem to be an insurmountable barrier in the treatment of pain which limits the effectiveness of analgesic opioids, which is connected with, for instance, the development of tolerance, the resistance of neuropathic pain to analgesic opioids, so-called “paradoxical pain”, and side effects such as chronic constipation, dizziness, disorders of consciousness, cognitive impairment, or respiratory depression (particularly in the case of an overdose).

In clinical practice, in order to reduce opioid doses, and thereby to avoid the risk of deleterious side effects, attempts have been made to introduce co-analgesics, which enhance the analgesic activity of opioid like drugs that block NMDA receptors, or drugs that stimulate the GABA-ergic system.

Magnesium (Mg) (II) is a physiological antagonist of NMDA receptors. Furthermore, as previously reported, Mg sulfate administered intraperitoneally in relatively low doses markedly potentiated opioid antinociception in different kinds of pain e.g., acute pain, neuropathic pain [2, 3]. It follows that the combined administration of opioid and Mg may in the future create very valuable therapeutic possibilities. Moreover, the concomitant oral administration of these compounds would hypothetically allow the use of this combination in outpatient conditions.

It is well-known that Mg salts are generally absorbed from the gastrointestinal tract poorly and slowly [4]. Moreover, the results of many studies indicate that a particle size reduction by micronization may improve digestive absorption, and consequently the bioavailability of various compounds [5]. Unfortunately, there is no available data comparing the rate and degree of digestive absorption, and the clinical efficacy of the micronized and the conventional form of Mg salts. Therefore, in the current study we investigated how various Mg salts (e.g. lactate dihydrate, hydroaspartate, chloride) in micronized form administered via the gastric tube, modify opioid antinociception in comparison with the common form of Mg salts in rats. The intestinal absorption of Mg salt in micronized and normal forms in the porcine gut sac model was also investigated.

## Materials and Methods

### Animals

The study was conducted according to the guidelines of the Ethical Committee for Experiments on Small Animals, Medical University of Warsaw, and adhered to guidelines published in the European directive 2010/63/EU on the protection of animals used for scientific purposes. The aforementioned Committee at the Medical University of Warsaw approved the experiment protocols (Permit no. 8/2011). For behavioral studies, male Wistar rats (250–300 g) were housed in a room maintained at a temperature of 22 ± 2°C under 12 h-12 h light–dark cycles. The animals had free access to food and water. Experimental groups consisted of six rats. For in vitro studies, pigs (180–200 kg) fasted 24 h before experiment were conducted.

### Drugs

Morphine sulfate was purchased from Warszawskie Zakłady Farmaceutyczne, oxycodone hydrochloride from Mundipharma GmbH, tramadol hydrochloride from Saneca Pharmaceuticals, Mg lactate dihydrate from LEK-AM Sp. Z o.o., Mg chloride from Chempur, Mg aspartate dihydrate from LGC Standards Sp. Z.o.o.. Ringer's components—sodium chloride, potassium chloride, calcium chloride were purchased from Chempur, while natrium lactate from L.G.C.

### Preparation and characterization of Mg micronized forms

The micronization of Mg salts was carried out in a Laboratory air-mill at the Pharmaceutical Research Institute. Pulverization was performed in the central chamber of the mill, jet energy mill as the process material was driven at near sonic velocity around the perimeter of the chamber by multiple jets of air. Importantly, no grinding media was involved. Size reduction was the result of the high-velocity collisions between particles of the process material itself. The interior of the chamber was designed to allow recirculation of over-sized particles, enhancing the incidence and the effect of these collisions. As particles were reduced in size and progressively lost mass, they naturally migrated toward the central discharge port. The micronized form of Mg salts used in experiments contained particles of Mg compounds of size D_90_<50 μm. The normal form of Mg salts used in experiments contained particles of sizes commonly available on the market.

### Drugs administration and doses

In behavioral studies morphine at a dose of 15 mg kg^-1^, tramadol at a dose of 125 mg kg^-1^, and oxycodone at a dose of 5 mg kg^-1^ were dissolved in distilled water. Mg lactate dihydrate and Mg hydroaspartate (in micronized and conventional form)–at a dose of 15 mg Mg ions kg^-1^ were suspended in 0.5% solution of methylcellulose. Mg chloride (in micronized and normal form)–at a dose of 15 mg Mg ions kg^-1^ was dissolved in distilled water. Control animals received a 0.5% solution of methylcellulose and distilled water respectively, according to the same time schedule. Of note, doses of opioids and Mg salts were selected experimentally, so that the separately administered investigated drug did not reveal significant analgesic action. Mg salts were administered before investigated opioids.

In studies of transport across porcine intestines *in vitro*, Mg lactate dihydrate [in micronized and normal form, at doses of 7.5 mg or 15 mg Mg ions for the intestinal sac] was suspended in 10 ml of 0.5% solution of methylcellulose. Additionally, in control samples a 0.5% solution of methylcellulose was placed in the intestinal sac.

### Gut sac preparation and intestinal permeation assay

The *in vitro* absorption studies of both micronized and normal forms of Mg salts were evaluated using a non-everted gut sac model, according to the method described by Ruan *et al*. [6] with slight modifications. Briefly, freshly isolated pieces of porcine intestine (jejunum) were obtained from a local slaughterhouse on the day of the experiments. The intestine was washed three times with Ringer lactate solution and then transported to the laboratory in cooled (4°C) and oxygenated Ringer lactate solution. The intestine was cut into pieces of 10 cm. The pieces of intestine were tied up at one end and incubated in Ringer lactate solution heated to 36°C for 30 min. 10 ml of Mg lactate dihydrate (micronized or normal form) suspension was placed in the intestine and then the other open end of the intestine was also tied to form a sac [7]. The filled sacs were immersed in a container filled with 100 ml of Ringer lactate solution and mixed very smoothly [8]. Importantly, the container was equipped with a water jacket with a capacity of 250 ml and connected to two thermostats. Each container was additionally equipped with a magnetic stirrer, a thermometer, and a gas mixture delivery end (oxygen/carbon dioxide, 95:5). Samples of 1 ml each were withdrawn from the serosal side at 15, 30, 45, 60 and 120 min, whereas the volume of the incubate was complementing each time with Ringer lactate solution. Ionized fraction of Mg makes up 67% of total Mg and actively influences functions of over 300 enzymes and nucleic acids [9, 10]. Therefore, in this study we determined ionized fractions of Mg using the potentiometric method by means of ion-selective electrodes, and clinical Analyzer Microlyte 6 (KONE, Finland). In the first stage, the examination of electrodes was performed. The analyzer automatically performed a calibration using three standard solutions. The moment the calibration was completed, the control serum (Nortrol, Thermo, Finland) was applied three times in order to validate the working electrodes. Under the above-mentioned conditions, the analyzer automatically converted the measured potential of the ion-selective electrode into a concentration of ionized Mg. Every four hours another calibration was carried out, as well as the exchange of standard solutions, and new calibration solutions measurements [9, 10]. The permeability of Mg ions across the intestine was calculated as a mean value from 10 samples (mmol of Mg ions/1l of Ringer solution).

### Behavioral studies

The acute activity of the investigated drugs was measured in short time periods (from 5 to 300 min) after a single administration. Prolonged activity of the investigated drugs was measured on the following day after initial administration, but before the next administration, as well as after the cessation of administration. In this case, drugs were administered for seven consecutive days (administration of morphine, oxycodone) or for ten days (administration of tramadol). All drugs were administered p.o.. However, in practice drugs were administered intragastrically via a gastric tube.

### Measurement of the nociceptive threshold

Changes in pain thresholds were determined using mechanical stimuli—the modification of the classic paw withdrawal test described by Randall and Selitto [11]. In order to produce a mechanical stimulation, progressively increasing pressure was applied to the dorsal surface of the rat’s paw using an analgesimeter (type 7200, Ugo-Basile Biological Research Apparatus, Comerio-Varese, Italy). The instrument used increased the force on the paw at a rate of 32 grams/second. The nociceptive threshold was defined as force in grams, at which the rat attempted to withdraw its right hind paw; so values of pressure were recorded at this very moment. At least two observers controlled the response.

The nociceptive thresholds (average of two trials) measured for each animal before a single administration of the investigated drugs (measurement of an acute activity) or in day 0 before the first administration (measurement of prolonged activity) constituted the baseline pain threshold—A. Next, measurements of the withdrawal threshold to mechanical stimulus were performed at 5, 15, 30, 60, 90, 120, 180, 240 and 300 min post drug-administration (measurements of an acute activity), or every day before drug administration (measurements of prolonged activity)—B. In all experimental sessions (i.e. every day, each drug investigated) the values of obtained thresholds (B) were compared to the baseline (A). Additionally, changes in pain threshold were calculated as a percent of the baseline value according to the following formula:

$$C=\left(\frac{B}{A}*100\%\right)-100\%$$

C: % of analgesia; A: pressure (in g), baseline pain threshold; B: pressure (in g) in consecutive measurements

Percentages of analgesia values calculated as above for individual animals were subsequently used to calculate average values in particular experimental groups and for statistical analysis.

### Statistical analysis

The results are expressed as mean values ± standard deviation of the mean (± S.E.M.). In behavioral studies, the statistical significance of differences between groups was evaluated by Student’s t-test (p ± 0.05 was accepted as statistically significant) and in *in vitro* studies by analysis of variance. In cases of significant differences in specific groups (divided according to Mg dose), the Games-Howell post-hoc test was used. All statistical calculations were conducted with the package of IBM SPSS Statistics 22.

## Results

### Behavioral studies

**Fig 1:**
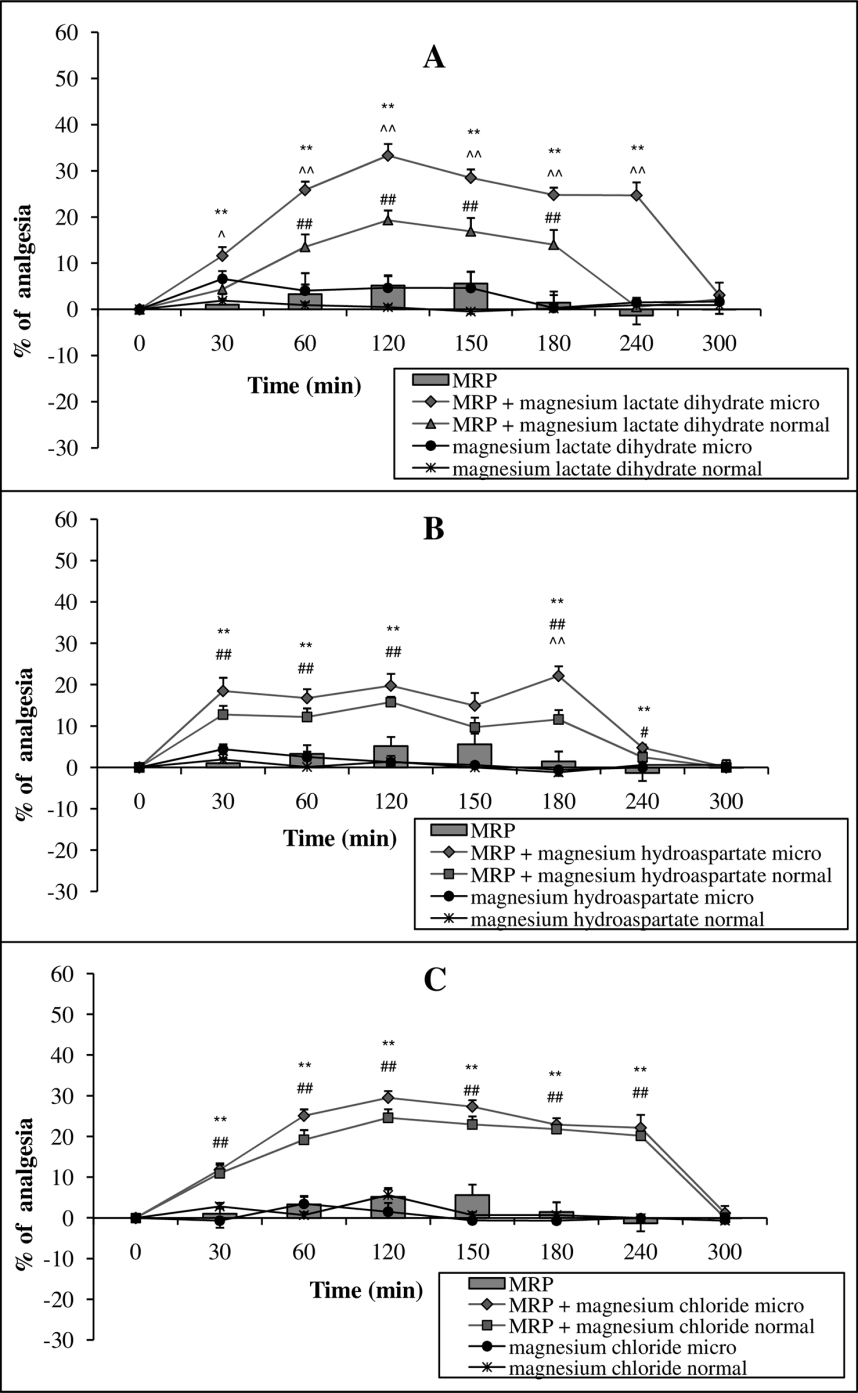
**The effect of magnesium salts (lactate dihydrate—A, hydroaspartate—B, chloride—C) in micronized form (micro) and a normal form at a dose of 15 mg magnesium ions kg**^**-1**^ **body mass (p.o.) on the analgesic activity of morphine (MRP) at a dose of 15 mg kg**^**-1**^ **body mass (p.o.).** Values are means ± SEM; **p < 0,01 MRP vs. MRP + micronized magnesium compound; ^##^p < 0,01, ^#^p < 0,05 MRP vs. MRP + normal magnesium compound; ^^^^p < 0,01, ^^^p < 0,05 MRP + micronized magnesium compound vs. MRP + normal magnesium compound.

A single administration of a micronized or normal form of Mg lactate dihydrate, or Mg hydroaspartate at a dose of 15 mg Mg ions kg^-1^ body mass (p.o.) with morphine at a dose of 15 mg kg^-1^ body mass (p.o.), significantly enhanced morphine-induced antinociception (Fig 1A and 1B). The activity of micronized forms of Mg lactate dihydrate and hydroaspartate was not only marked but also appeared earlier (from 30 min) and lasted longer (to 240 min for Mg lactate dihydrate or to 180 min for Mg hydroaspartate, respectively), as compared to the conventional form of Mg salts (from 60 to 180 min for Mg lactate dihydrate or from 30 to 180 for Mg hydroaspartate, respectively). On the other hand, the administration of equivalent doses of micronized or conventional Mg chloride (15 mg of Mg ions kg^-1^ body mass, p.o.) enhanced morphine-induced antinociception in a similar manner (Fig 1C). Importantly, neither morphine nor Mg lactate dihydrate, hydroaspartate, and chloride administered alone in a micronized or normal form showed any analgesic activity (Fig 1, Table A in S1 File).

**Fig 2:**
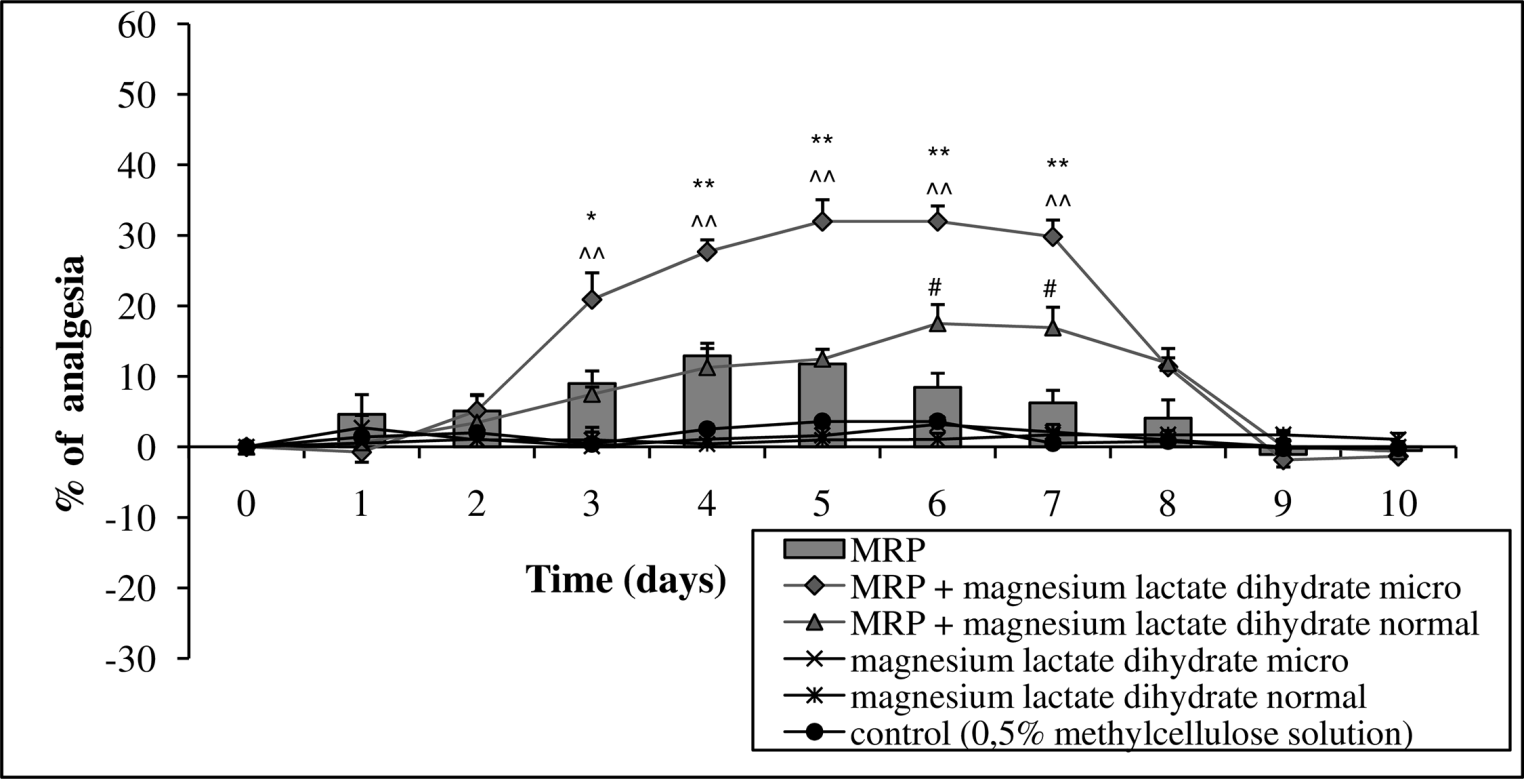
The effect of long-term, 7-day administration of magnesium lactate dihydrate in micronized form (micro) or normal form (15 or 30 mg magnesium ions kg^-1^ body mass [p.o.], respectively) on the analgesic activity of morphine (MRP, 15 mg kg^-1^ body mass, p.o.). Day 0 –measurement of the initial pain threshold and the first day of administration of the tested compounds, days 1–7 –measurement of the prolonged activity of the tested drugs, days 8–10 –measurement following the cessation of administration. Values are means ± SEM; **p < 0,01, *p < 0,05 MRP vs. MRP + micronized magnesium compound; ^#^p < 0,05 MRP vs. MRP + normal magnesium compound; ^^^^p < 0,01 MRP + micronized magnesium compound vs. MRP + normal magnesium compound.

A 7-day premedication with micronized Mg lactate dihydrate (15 mg Mg ions kg^-1^ p.o.) also resulted in an enhancement of the analgesic activity of morphine (15 mg kg^-1^ body mass, p.o.). This activity began on the third day of the measurement and increased gradually to day 7. Cessation of drug administration caused a gradual return to the baseline values. The activity of normal Mg lactate dihydrate on morphine antinociception was much weaker and appeared only on days 6 and 7 of the measurements, however this effect was not significant. The separate administration of morphine slightly increased the threshold for mechanical nociceptive stimuli, whereas the separate administration of Mg lactate dihydrate in the micronized form or conventional form did not alter the pain threshold (Fig 2, Table D in S1 File).

**Fig 3:**
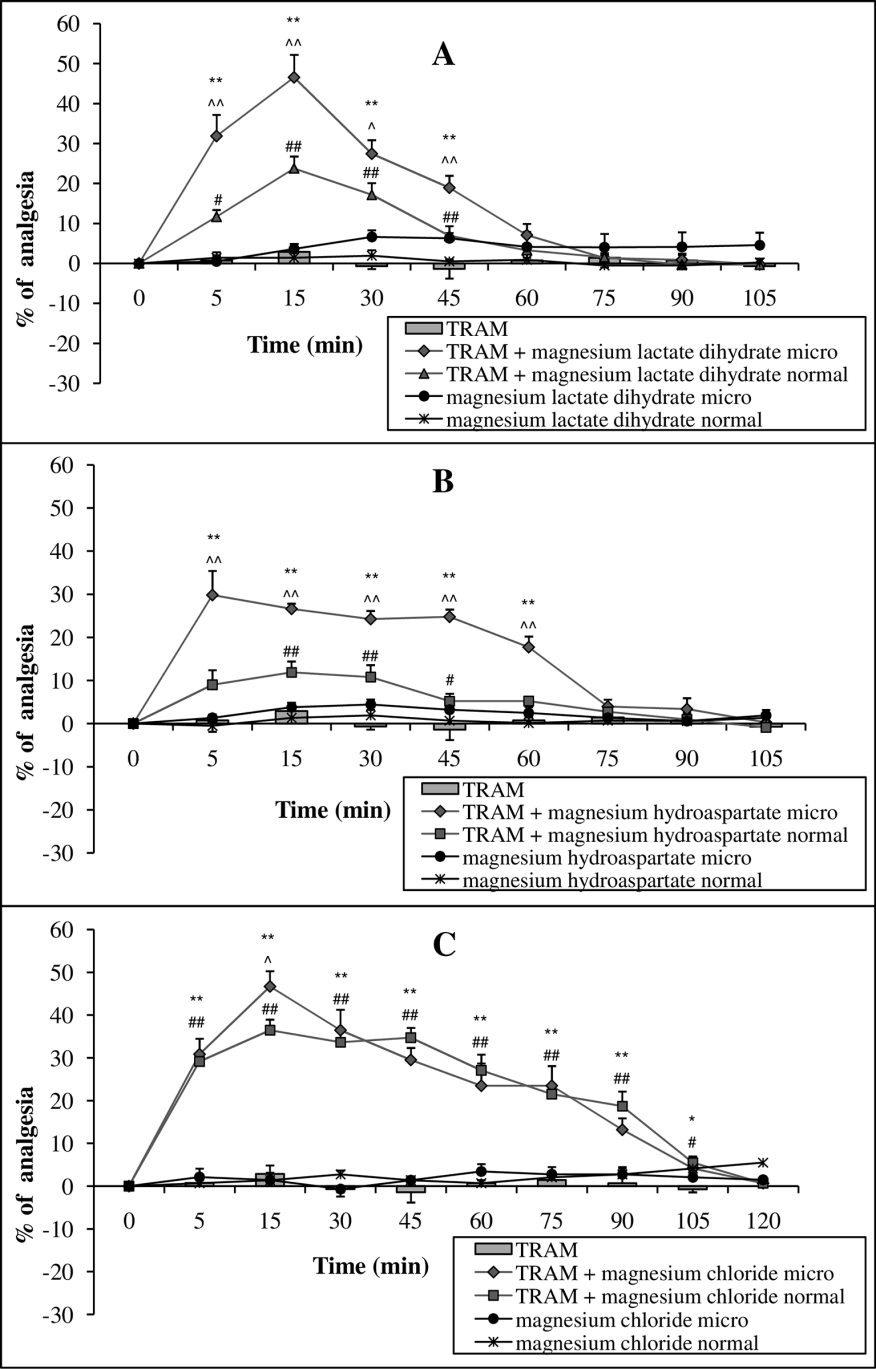
**Effect of magnesium salts (lactate dihydrate—A, hydroaspartate—B, chloride—C) in micronized form (micro) and normal form at a dose of 15 mg magnesium ions kg**^**-1**^ **body mass (p.o.) on the analgesic activity of tramadol (TRAM) at a dose of 125 mg kg**^**-1**^ **body mass (p.o.).** Values are means ± SEM; **p < 0,01, *p < 0,05 TRAM vs. TRAM + micronized magnesium compound; ^##^p < 0,01, ^#^p < 0,05 TRAM vs. TRAM + normal magnesium compound; ^^^^p < 0,01, ^^^p < 0,05 TRAM + micronized magnesium compound vs. TRAM + normal magnesium compound.

A single administration of the micronized or conventional form of Mg lactate dihydrate and Mg hydroaspartate at a dose of 15 mg Mg ions kg^-1^ p.o., increased the antinociceptive activity of tramadol (125 mg kg^-1^ body mass, p.o.). The enhancement of opioid-induced antinociception by Mg already appeared at 5 min post-administration, with the maximum activity at 15 min and lasted until 45 min (for Mg lactate dihydrate) or at 5–60 min (for Mg hydroaspartate). Activity of both conventional forms of Mg on tramadol antinociception was weaker than the micronized forms (Fig 3A and 3B). Additionally, the administration of equivalent doses of Mg chloride in micronized or conventional forms (15 mg Mg ions kg^-1^ body mass, p.o.) with tramadol (125 mg kg^-1^ body mass, p.o.) enhanced tramadol antinociception to the same extent (Fig 3C). The maximal pain-relieving action, calculated as the % of analgesia, for both micronized forms of Mg lactate dihydrate and Mg chloride was reached at 5 min post-administration, and was equal. Importantly, tramadol administered separately did not exhibit analgesic activity in the presented pain model (Fig 3, Table B in S1 File).

**Fig 4:**
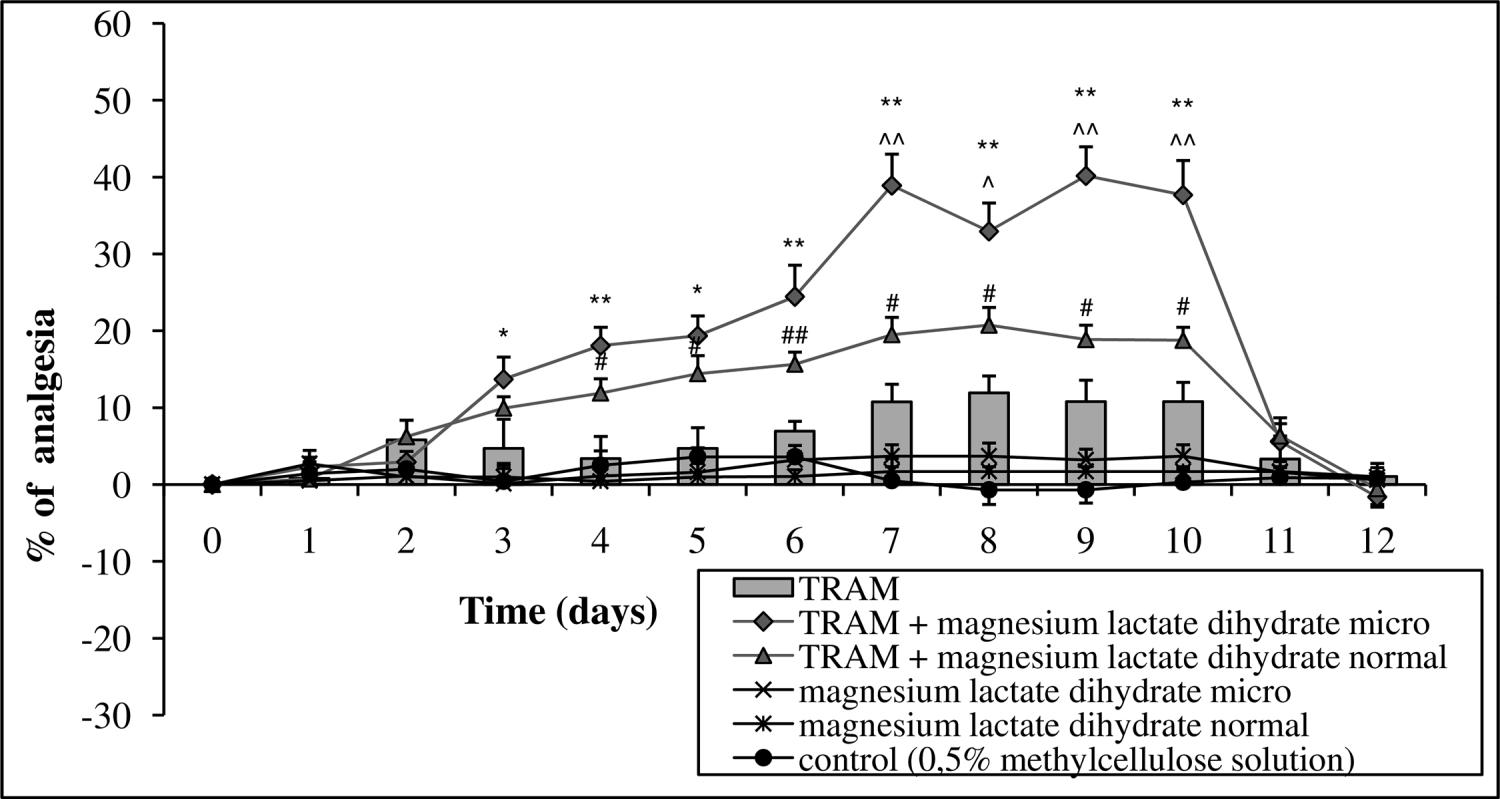
The effect of long-term 10-day administration of magnesium lactate dihydrate in micronized (micro) form or normal form (15 or 30 mg magnesium ions kg^-1^ body mass [p.o], respectively) on the analgesic activity of tramadol (TRAM, 125 mg kg^-1^ body mass, p.o.). Day 0 –measurement of the initial pain threshold and the first day of administration of tested compound, days 1–10 –measurement of the prolonged activity of the tested drugs, days 11–12 –measurement following the cessation of administration. Values are means ± SEM; **p < 0,01, *p < 0,05 TRAM vs. TRAM + micronized magnesium compound; ^##^p < 0,01, ^#^p < 0,05 TRAM vs. TRAM + normal magnesium compound; ^^^^p < 0,01, ^^^p < 0,05 TRAM + micronized magnesium compound vs. TRAM + normal magnesium compound.

Micronized Mg lactate dihydrate (15 mg Mg ions kg^-1^ body mass, p.o.) administered with tramadol for 10 consecutive days (125 mg kg^-1^ body mass, p.o.) enhanced the long-term analgesic activity of the examined opioid. This effect began on the fourth day, gradually increased to day 7, and was then maintained to day 10 of the measurements. After the removal of the tested substances, the pain threshold returned to control values by day 11 of the measurements. The activity of conventional Mg lactate dihydrate was similar to the micronized form, up to day 6 of the measurements, and then significantly weaker. The separate administration of tramadol raised the threshold for mechanical nociceptive stimuli from days 7 to 10 of the measurements (Fig 4, Table E in S1 File).

**Fig 5:**
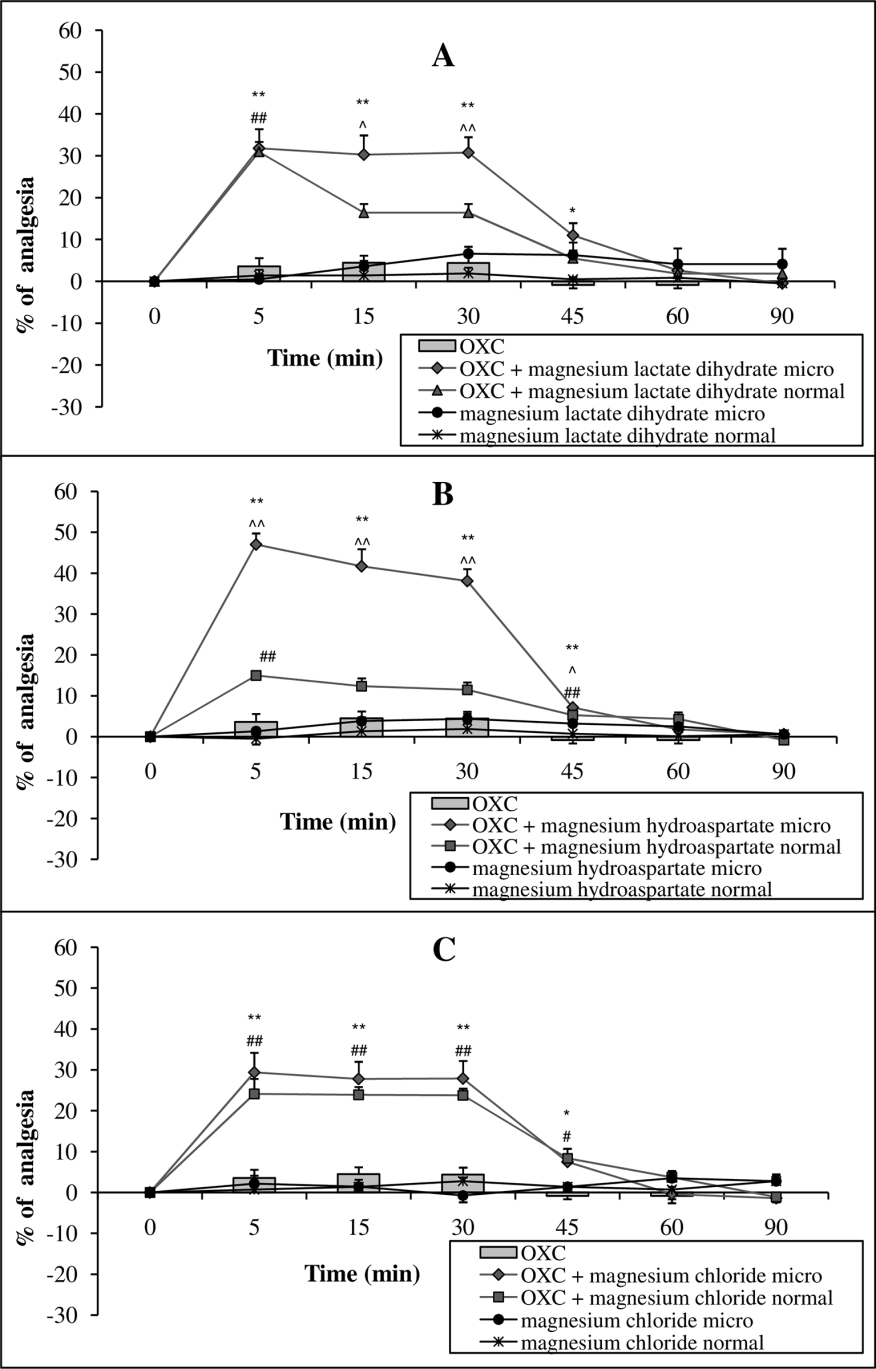
**The effect of magnesium salts (lactate dihydrate—A, hydroaspartate–B, chloride—C) in micronized form (micro) and normal form at a dose of 15 mg magnesium ions kg**^**-1**^ **body mass (p.o.) on the analgesic activity of oxycodone (OXC) at a dose of 5 mg kg**^**-1**^ **body mass (p.o.).** Values are means ± SEM; **p < 0,01, *p < 0,05 OXC vs. OXC + micronized magnesium compound; ^##^p < 0,01, ^#^p < 0,05 OXC vs. OXC + normal magnesium compound; ^^^^p < 0,01, ^^^p < 0,05 OXC + micronized magnesium compound vs. OXC + normal magnesium compound.

A single administration of the micronized or conventional form of Mg lactate dihydrate or Mg hydroaspartate at a dose of 15 mg of Mg ions kg^-1^ body mass (p.o.) resulted in a significant enhancement of oxycodone-induced antinociception (5 mg kg^-1^ body mass, p.o.). The activity appeared already at 5 min, and it was maintained at a constant level up to 30 min of the study, then became weaker at the time-point of 45 min, and disappeared at 60 min. The activity of the normal form of Mg lactate dihydrate on oxycodone antinociception reached its maximum at 5 min, then slightly decreased and remained at the same level for the next 15 min (from time-point of 15 to 30 min); for Mg hydroaspartate, significance was observed only at 5 min, following the administration of the opioid (Fig 5A and 5B). The administration of equivalent doses of Mg chloride (15 mg Mg ions kg^-1^ body mass, p.o.) with oxycodone (5 mg kg^-1^ body mass, p.o.) also enhanced oxycodone-induced antinociception; however, no differences in the strength of action between both forms were observed (Fig 5C). The administration of oxycodone alone showed no analgesic activity (Fig 5, Table C in S1 File).

**Fig 6:**
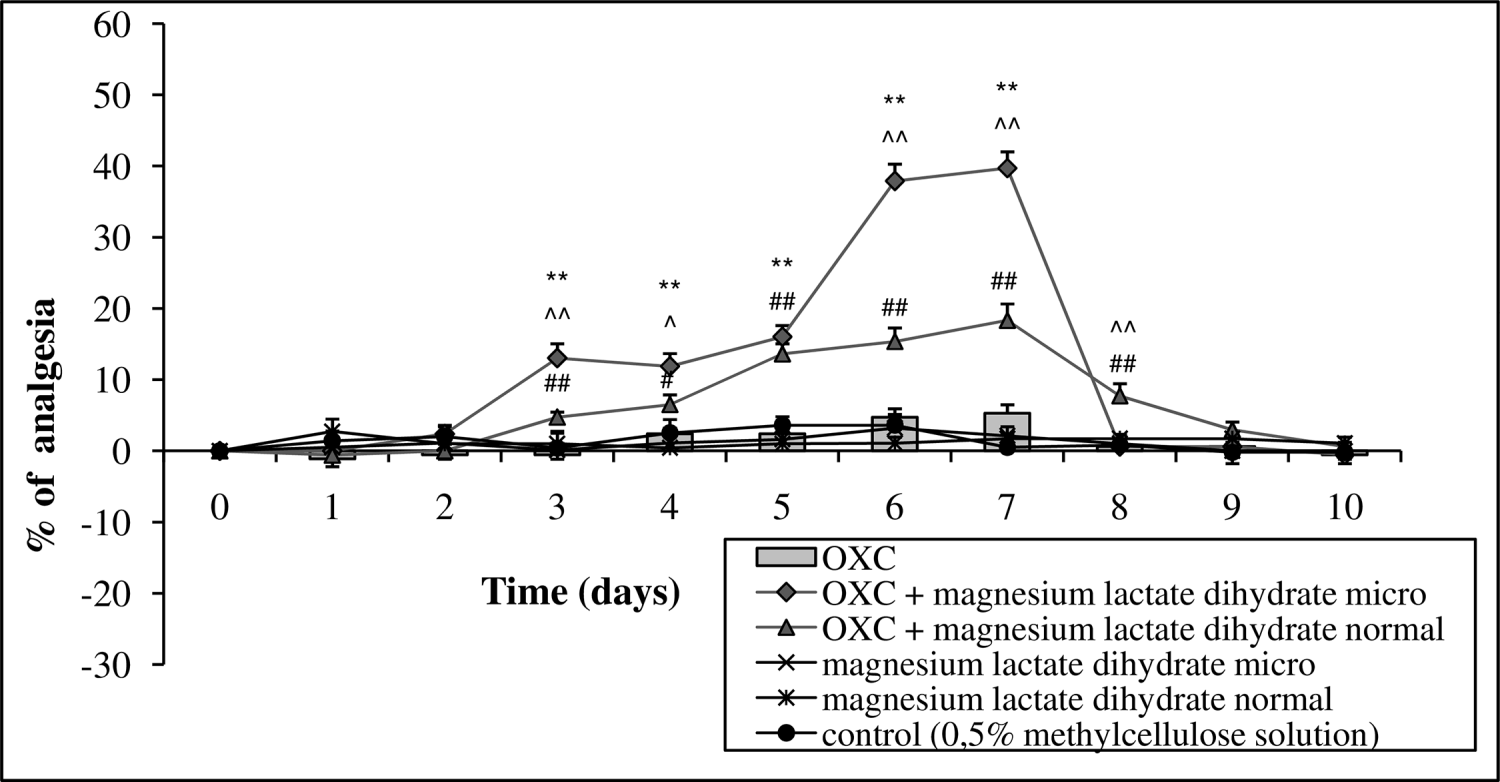
The effect of long-term 7-day administration of magnesium lactate dihydrate in micronized (micro) or normal form (15 or 30 mg of magnesium ions kg^-1^ body mass [p.o.], respectively) on the analgesic activity of oxycodone (OXC, 5 mg kg^-1^ body mass, p.o.). Day 0 –measurement of the initial pain threshold and the first day of administration of tested compound, days 1–7 –measurement of the prolonged activity of the tested drugs, days 8–10 –measurement following the cessation of administration. Values are means ± SEM; **p < 0,01 OXC vs. OXC + micronized magnesium compound; ^##^p < 0,01, ^#^p < 0,05 OXC vs. OXC + normal magnesium compound; ^^^^p < 0,01, ^^^p < 0,05 OXC + micronized magnesium compound vs. OXC + normal magnesium compound.

The micronized form of Mg lactate dihydrate (15 mg Mg ions kg^-1^ body mass, p.o.) administered for 7 consecutive days together with oxycodone (5 mg kg^-1^ body mass, p.o.), enhanced the long-term analgesic activity of the examined opioid. The activity began on the third day of measurement and reached its peak on days 6 and 7 of the study. One day after the removal of the tested substances, a rapid return of the pain threshold to control values was observed. The activity of normal Mg lactate dihydrate was similar in the initial phase of the treatment (to day 5), where after it became weaker compared to the micronized form. The separate administration of oxycodone did not exhibit significant analgesic activity (Fig 6, Table D in S1 File).

### In vitro studies of the transport across porcine intestine

**Fig 7:**
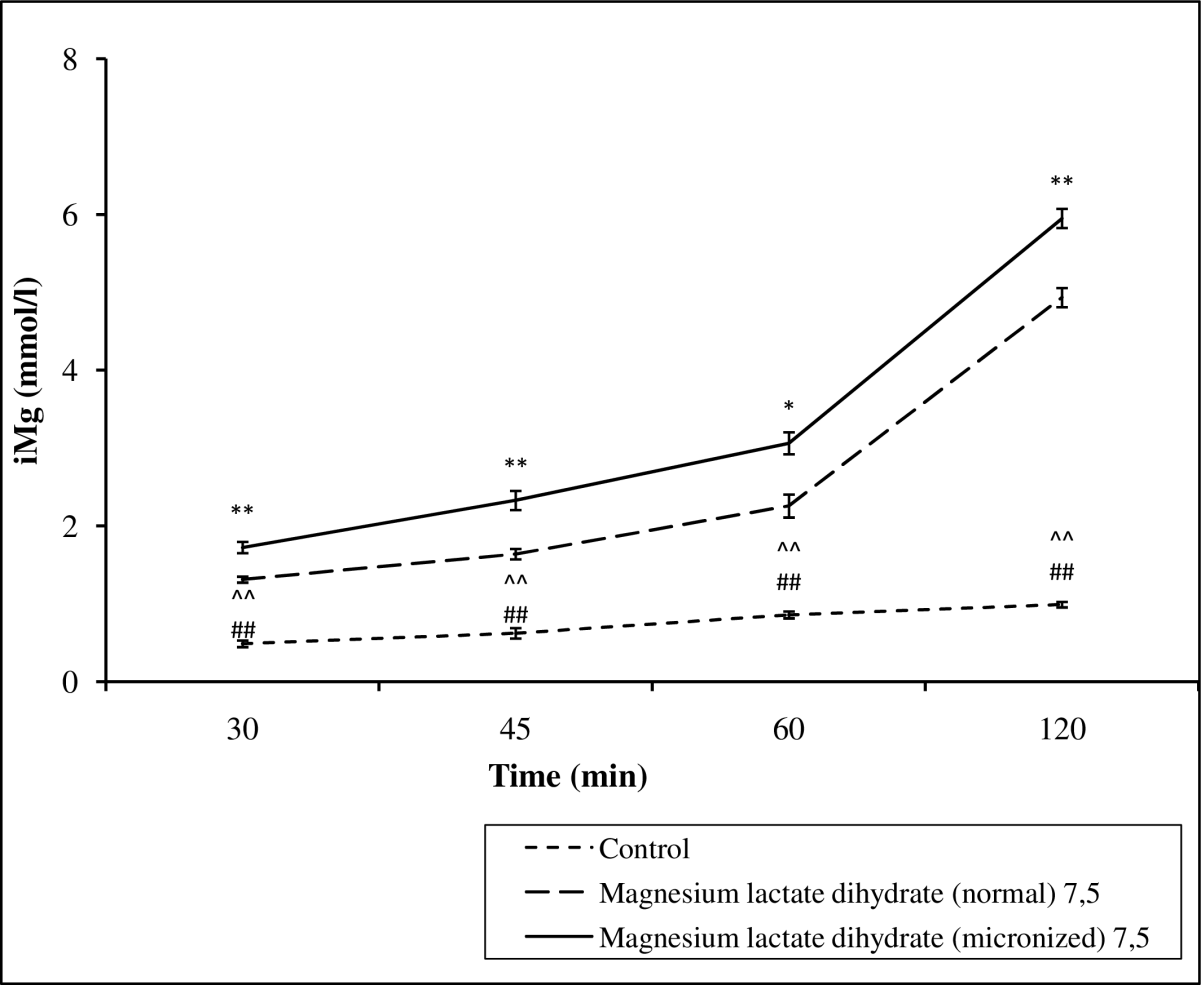
The effect of magnesium lactate dihydrate in normal and micronized forms at doses of 7.5 mg kg^-1^ of magnesium ions kg^-1^ body mass on the concentration of ionized fraction of magnesium. Values are means ± SEM; ^**^p < 0,01, *p < 0,05 normal magnesium lactate dihydrate vs magnesium micronized lactate dihydrate; ^##^p < 0,001 control vs normal magnesium lactate dihydrate; ^^^^p < 0,001, control vs micronized magnesium lactate dihydrate.

**Fig 8:**
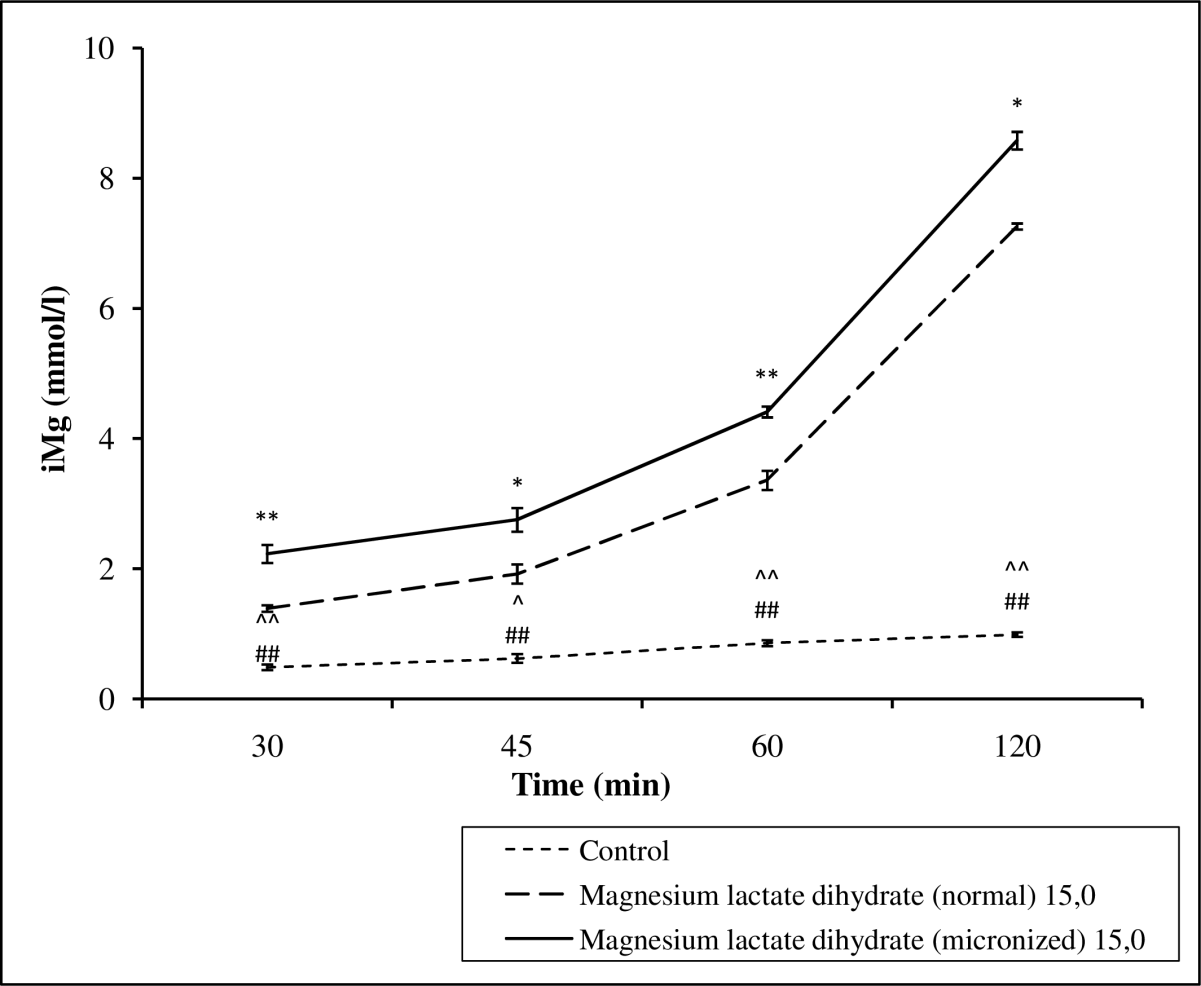
The effect of magnesium lactate dihydrate in normal and micronized forms at doses of 15 mg kg^-1^ of magnesium ions kg^-1^ body mass on the concentration of ionized fraction of magnesium. Values are means ± SEM; **p < 0,001, *p < 0,01 normal magnesium lactate dihydrate vs magnesium micronized lactate dihydrate; ^##^p < 0,001 control vs normal magnesium lactate dihydrate; ^^^^p < 0,001, ^^^p < 0,05 control vs micronized magnesium lactate dihydrate.

Mg lactate dihydrate administered in micronized form at a dose of 7.5 or 15 mg ions/sample was absorbed from the intestines to a greater extent in comparison to the normal form of Mg ions, while the concentration slightly increased also in the control group (Figs 7 and 8, Table F in S1 File).

## Discussion

In this study, we have demonstrated for the first time that a micronization, and per oral administration of organic magnesium salts (lactate dihydrate, hydroaspartate) with opioid (p.o.), increases opioid antinociception to a significantly greater extent than conventional forms of magnesium salts in rats. It is important to emphasize that the administration of equivalent doses of Mg chloride (15 mg Mg ions kg^-1^ body mass, p.o.) with opioids (p.o.) also enhanced the analgesic activity of opioid, but there is no difference in the strength of action between both forms.

Research on the interaction of opioids and Mg salts with regards to analgesic action date back several years, but the amount of available literature is relatively limited. However, according to the information available it can be said that a co-administration of Mg and opioids can be clinically useful [12]. This is proven by the studies of Özalevli *et al*. [13], who demonstrated that intrathecal addition of Mg sulfate to bupivacaine and fentanyl, prolonged the period of postoperative spinal analgesia without additional side effects. Also Buvanendran *et al*. [14] suggested that for patients receiving spinal analgesia for labour, the addition of Mg sulfate to the fentanyl resulted in prolonged analgesia, whereas no increase in side effects was noted. Although these beneficial effects were observed in patients with postoperative pain, the results from studies on animals indicated that e.g., Mg sulphate administered parenterally may potentiate the analgesic effects of opioids also in different types of acute and chronic pain [2, 15]. Similar action was also found in neuropathic pain states [3, 16, 17]. Moreover, in animal studies, Mg delayed the development of tolerance to the analgesic effect of morphine, and reduced the symptoms of withdrawal in morphine dependence [18]. All these reports improved the image of Mg as a great adjuvant to opioid therapies, despite the fact that only its parental administration was noticed. However, it seems obvious that the administration of opioids and Mg salts orally instead of parenterally would facilitate a widespread use of the synergistic analgesic effect of opioid and Mg by patients in outpatient conditions.

The results presented here is the second manuscript available that describes oral administration of Mg and opioids. Similarly, to Suresh *et al*. [19] the treatment with Mg resulted in an increase of analgesic activity of opioids in small doses. Importantly, in our study this enhancement of the pain-relieving action exerted by morphine, tramadol, and oxycodone was observed for each type of the Mg salt used (i.e. lactate dihydrate, hydroaspartate, chloride). Of note, no analgesic effect was seen for mentioned opioids (when given alone) in a model of acute pain after mechanical stimuli. In addition to this, due to poor absorption of Mg from common salts in the gastrointestinal tract, we decided to administer micronized forms of these salts. Although in preclinical and clinical studies it was observed that organic Mg salts (e.g. acetate, pidolate, citrate, gluconate, lactate, aspartate) are better sources of Mg itself than inorganic Mg salts (MgO, MgCl_2_, MgSO_4_, MgCO_3_) [20]; and a small analgesic inactive dose of two organic salts (i.e. Mg lactate dihydrate and hydroaspartate) and one inorganic salt (i.e. Mg chloride) were chosen for further analysis.

As mentioned above, organic but not nonorganic Mg salts administered p.o. in micronized forms intensified to a greater extent the analgesic effects of opioids (p.o.) than salts in the conventional forms.

There are at least three possible explanations why a micronized form of Mg salts enhanced the analgesic action of the opioid more markedly than unmicronized preparations of this cation. Micronization can (i) increase or (ii) accelerate absorption of Mg from the gastrointestinal tract, but it can also (iii) change the ratio of the ionized to unionized form of absorbed Mg.

In fact, it is well known that the physicochemical properties of drugs are decisive for clinical efficacy, and consequently lead to therapeutic success. Micronization seems to be one of the methods of enhancing the rate of dissolution and bioavailability of various compounds with poor water solubility [5, 21, 22]. According to the Noyes-Whitney equation, the rate of dissolution depends on the effective surface area of the drug particles [23]. Moreover, the solubility increases exponentially as a function of particle size [24].

It seems to be crucial since, as Khan *et al*. [21] reported, almost 90 percent of drugs approved after 1995 are characterized by poor solubility, poor permeability, or both. Nowadays, more and more compounds are included into class II of the Biopharmaceutical Classification System, which means that their bioavailability is limited especially by their rate of solvation. Furthermore, improving water solubility seems to be essential also due to the characteristics of the gastrointestinal tract and its specific water-dependent absorption mechanism [21].

In 1980, for the first time, the micronized form of progesterone was launched for sale [25, 26]. Later investigations also indicated that particle size reduction distinctly increased the bioavailability of compounds with low water solubility such as digoxin, danzol, aspirin, glisentide, tibolon, fenofibrate, cefpodoxime, resveratrol etc. [27–39].

It is interesting to note that in this study, a potentiating of opioid antinociception was found to be stronger for the micronized forms of Mg salts that are found to be poorly soluble in water (lactate dihydrate and hydroaspartate) than for the normal form. On the contrary, Mg chloride administration resulted in no significant differences in the strength of action observed for both forms. However, this observation is not surprising, because Mg chloride has good solubility in water, so the dimension of its particles theoretically should not affect the absorption.

Thus, we can also speculate that micronization might accelerate the absorption of poorly soluble in water Mg salts from the gastrointestinal tract. Indeed, in the current study a micronized form of Mg more rapidly increased an opioid-induced antinociception than a conventional form. This in turn can indirectly indicate that the micronized form of Mg salts are more rapidly absorbed from the gastrointestinal tract. Unfortunately, in the intestinal absorption of Mg lactate dihydrate in micronized and normal forms in the porcine gut sac model we were not able to demonstrate a faster absorption of the micronized form in comparison with the conventional form. However, micronized Mg lactate dihydrate administered both at a dose of 7.5 mg or 15 mg Mg ions/sample was absorbed to a greater extent than conventional form. Interestingly, the concentration of Mg ions increased also in the control group, which was probably due to the release of the Mg ions from the animal tissue.

It can also be hypothesized that the micronization of Mg can change the proportion of the absorption of ionized/unionized Mg. Altura *et al*. [40] showed that diets enriched with different oral formulations of Mg given for 6 days resulted in significant elevations in serum ionized Mg and % ionized Mg, but not total Mg in male volunteers. The authors suggested that no change in total Mg through diet may not reflect changes in the biologically-active Mg. We are not able to confirm this hypothesis because in this study we decided to determine only ionized Mg.

## Conclusions

Mg salts (i.e. lactate dihydrate, hydroaspartate chloride) administered p.o. in a small dose increased analgesic activity of small doses of opioids. This effect is significantly stronger for a micronized form of Mg lactate dihydrate and hydroaspartate but not Mg chloride compared to common forms, which is probably associated with a greater absorption of micronized salts of Mg from the intestines.

## Supporting Information

S1 File Supporting data for the manuscript. This file contains Tables: A, B, C, D, E, F.
(DOCX)
