# Supplementary material for: Micronized Organic Magnesium Salts Enhance Opioid Analgesia in Rats
Source: PLoS One. 2016 Oct 28;11(10):e0161776. doi: 10.1371/journal.pone.0161776 (PMC5085085; doi:10.1371/journal.pone.0161776)
Supplement: S1 File — This file contains Tables: A, B, C, D, E, F. (DOCX) [file pone.0161776.s001.docx]

**SUPPLEMENTARY TABLES**

| **Compound** | **30’** | **60’** | **120’** | **150’** | **180’** | **240’** | **300’** |
| --- | --- | --- | --- | --- | --- | --- | --- |
| MRP | 1±3,52  Me=0 | 3,27±5,57  Me=4,3 | 5,18±5,77  Me=6,3 | 5,61±6,91  Me=6,7 | 1,46±6,3  Me=1 | -1,36±4,96  Me=-1,1 | -0,07±2,4  Me=0 |
| MRP + magnesium lactate micro | 11,6±5,71  Me=12,5 | 25,88±5,36  Me=27,6 | 33,31±7,4  Me=33,3 | 28,51±5,4  Me=30,4 | 24,77±4,81  Me=25 | 24,71±8,39  Me=25 | 3,09±7,98  Me=0 |
| MRP + magnesium lactate normal | 4,37±4,34  Me=4,15 | 13,52±6,51  Me=14,5 | 19,3±5,05  Me=20 | 16,88±7,2  Me=16,65 | 14,02±7,74  Me=11,3 | 0,53±0,83  Me=0 | 2,17±3,22  Me=0,8 |
| MRP + magnesium hydroaspartate micro | 18,5±7,89  Me=17,85 | 16,72±5,28  Me=17,85 | 19,77±7  Me=16,4 | 14,92±7,6  Me=14,3 | 22,17±5,92  Me=21,8 | 4,75±4,31  Me=5,35 | 0±0  Me=0 |
| MRP + magnesium hydroaspartate normal | 12,77±5,2  Me=12,7 | 12,17±5,19  Me=10,9 | 15,8±3,24  Me=16,4 | 9,72±5,65  Me=7,1 | 11,57±5,59  Me=9,1 | 2,47±3,02  Me=1,85 | 0±2,34  Me=0 |
| MRP + magnesium chloride micro | 11,8±4,72  Me=12,9 | 25,08±4,73  Me=24,1 | 29,49±5,2  Me=30,4 | 27,34±4,8  Me=25 | 22,91±4,7  Me=24,5 | 22,17±9,59  Me=18,8 | 1,14±5,37  Me=0 |
| MRP + magnesium chloride normal | 10,98±5,1  Me=10 | 19,2±5,88  Me=20 | 24,6±5,17  Me=25 | 23±4,94  Me=21,3 | 21,8±3,2  Me=21,3 | 20,18±5,17  Me=20,7 | 0,03±2,09  Me=0 |

**Table A. Descriptive statistics concerning the effect of magnesium salts (lactate, hydroaspartate, chloride) in micronized form (micro) and a normal form on the analgesic activity of morphine (MRP)**

| **Compound** | **15’** | **30’** | **45’** | **60’** | **75’** | **90’** | **105’** |
| --- | --- | --- | --- | --- | --- | --- | --- |
| TRAM | 2,88±4,58  Me=3,6 | -0,68±1,52  Me=0 | -1,4±5,37  Me=-3,4 | 0,72±1,61  Me=0 | 1,44±1,97  Me=0 | 0,7±3,9  Me=0 | -0,72±1,6  Me=0 |
| TRAM + magnesium lactate micro | 46,6±13,8  Me=48,45 | 27,42±8,36  Me=29,3 | 18,93±7,38  Me=19,25 | 7,1±6,84  Me=5,5 | 1,35±1,56  Me=0,9 | 0,9±3  Me=1,55 | -0,2±2,32  Me=0 |
| TRAM + magnesium lactate normal | 23,77±7,22  Me=25 | 17,17±7,08  Me=14,3 | 6,87±1,74  Me=7,1 | 3,28±3,63  Me=3,65 | 1,53±2,44  Me=0 | -0,28±2,91  Me=0 | -0,3±1,77  Me=0 |
| TRAM + magnesium hydroaspartate micro | 26,62±2,87  Me=27,25 | 24,23±4,55  Me=23,95 | 24,77±4,12  Me=25,65 | 17,7±6,19  Me=18,1 | 3,95±3,79  Me=3,6 | 3,37±6,03  Me=3,55 | 0,28±0,69  Me=0 |
| TRAM + magnesium hydroaspartate normal | 11,9±6,2  Me=14,3 | 10,73±6,9  Me=8,9 | 5,21±4,14  Me=5,35 | 5,2±2,5  Me=5,35 | 2,75±1,54  Me=3,6 | 0,95±2,28  Me=0 | -0,9±1,5  Me=0 |
| TRAM + magnesium chloride micro | 46,7±8,7  Me=45,85 | 36,48±11,7  Me=35,4 | 29,53±6,86  Me=29,15 | 23,5±12,69  Me=25 | 23,5±11,23  Me=25 | 13,22±6,69  Me=16,7 | 4,15±4,55  Me=4,15 |
| TRAM + magnesium chloride normal | 36,48±6,15  Me=38,55 | 33,68±6,24  Me=33,3 | 34,7±5,68  Me=33,3 | 27,08±9  Me=29,15 | 21,55±5,53  Me=20,85 | 18,75±8,22  Me=16,7 | 5,55±3,38  Me=6,25 |

**Table B. Descriptive statistics concerning the effect of magnesium salts (lactate, hydroaspartate, chloride) in micronized form (micro) and a normal form on the analgesic activity of tramadol (TRAM)**

| **Compound** | **5’** | **15’** | **30’** | **45’** | **60’** | **90’** |
| --- | --- | --- | --- | --- | --- | --- |
| OXC | 3,55±4,1  Me=3,55 | 4,45±3,39  Me=5,35 | 4,37±3,42  Me=5,2 | -0,82±1,65  Me=0 | -0,82±1,65  Me=0 | 0±0  Me=0 |
| OXC + magnesium lactate micro | 31,78±12,13  Me=28,6 | 30,27±12,06  Me=28,6 | 30,76±9,7  Me=32,1 | 11,01±7,72  Me=14,3 | 2,57±4,39  Me=0 | -0,47±1,24  Me=0 |
| OXC + magnesium lactate normal | 30,98±1,49  Me=31 | 16,4±1,33  Me=16,4 | 16,4±1,33  Me=16,4 | 5,5±1,2  Me=5,5 | 1,88±1,17  Me=1,9 | 1,88±1,17  Me=1,9 |
| OXC + magnesium hydroaspartate micro | 47,03±6,56  Me=50 | 41,67±10,28  Me=44,65 | 38,08±7,04  Me=35,7 | 7,1±0  Me=7,1 | 1,78±2,98  Me=0 | 0,6±1,47  Me=0 |
| OXC + magnesium hydroaspartate normal | 14,95±2,02  Me=14,3 | 12,4±3,8  Me=14,3 | 11,5±3,62  Me=12,5 | 5,25±1,91  Me=5,15 | 4,35±3,29  Me=5,15 | -0,82±1,65  Me=0 |
| OXC + magnesium chloride micro | 29,37±12,74  Me=25 | 27,83±11,04  Me=25 | 27,9±11,46  Me=27,6 | 7,46±4,32  Me=7,1 | -0,37±5,79  Me=0 | -1,43±2,63  Me=0 |
| OXC + magnesium chloride normal | 24,12±9,12  Me=28,6 | 23,93±4,55  Me=21,4 | 23,85±3,81  Me=22,25 | 8,43±5,67  Me=7,1 | 3,65±4  Me=3,55 | -1,12±2,73  Me=0 |

**Table C. Descriptive statistics concerning the effect of magnesium salts (lactate, hydroaspartate, chloride) in micronized form (micro) and a normal form on the analgesic activity of oxycodone (OXC)**

| Day | MRP | MRP + magnesium lactate micro | MRP + magnesium lactate normal | OXC | OXC + magnesium lactate micro | OXC + magnesium lactate normal |
| --- | --- | --- | --- | --- | --- | --- |
| 1 | 4,63±6,79  Me=3,35 | -0,77±3,42  Me=0 | 0,6±1,47  Me=0 | -1,12±2,73  Me=0 | 0±0  Me=0 | -0,6±1,47  Me=0 |
| 2 | 5,07±5,47  Me=5 | 5,13±5,63  Me=4,75 | 3,43±3,81  Me=2,9 | -0,55±1,35  Me=0 | 2,38±2,91  Me=1,8 | 0±2,28  Me=0 |
| 3 | 9±4,31  Me=8,35 | 20,9±9,33  Me=20,35 | 7,45±2,51  Me=7,4 | -0,55±1,35  Me=0 | 13,02±4,93  Me=12,5 | 4,73±1,76  Me=3,6 |
| 4 | 12,9±4,5  Me=11,65 | 27,68±4,14  Me=25,4 | 11,25±6,66  Me=7,4 | 2,4±1,86  Me=3,6 | 11,87±4,4  Me=12,5 | 6,48±3,4  Me=5,35 |
| 5 | 11,75±5,26  Me=11,65 | 31,97±7,56  Me=32,75 | 12,45±3,49  Me=13,05 | 2,38±2,91  Me=1,8 | 16,02±3,86  Me=16,1 | 13,62±3,52  Me=14,3 |
| 6 | 8,45±4,86  Me=8,35 | 31,98±5,46  Me=31,3 | 17,5±6,67  Me=18,7 | 4,7±2,92  Me=5,35 | 37,92±5,96  Me=39,3 | 15,37±4,73  Me=16,1 |
| 7 | 6,23±4,3  Me=5 | 29,82±5,78  Me=28,15 | 16,9±7,16  Me=18,7 | 5,23±2,92  Me=6,9 | 39,7±5,61  Me=41,1 | 18,32±5,66  Me=21,05 |
| 8 | 4,08±6,39  Me=3,35 | 11,35±3,13  Me=12 | 11,87±5,18  Me=14,85 | 0,6±1,47  Me=0 | 0,6±1,47  Me=0 | 7,7±4,21  Me=7,1 |
| 9 | -1,07±1,65  Me=0 | -1,85±2,82  Me=-0,8 | 0,1±4,68  Me=0 | 0,6±1,47  Me=0 | 0,6±1,47  Me=0 | 2,95±2,67  Me=3,5 |
| 10 | -0,52±2,44  Me=0 | -1,32±1,54  Me=-0,8 | -0,63±1,55  Me=0 | -0,55±1,35  Me=0 | -0,57±1,39  Me=0 | 0,6±1,47  Me=0 |

**Table D. Descriptive statistics concerning the effect of long-term administration of magnesium lactate in micronized form (micro) or normal form on the analgesic activity of morphine (MRP) and oxycodone (OXC)**

| **Day** | **TRAM** | **TRAM magnesium lactate micro** | **TRAM magnesium lactate normal** |
| --- | --- | --- | --- |
| 1 | 0,77±4,49  Me=0 | 2,25±3,08  Me=1 | 0,73±5,47  Me=1,9 |
| 2 | 5,8±6,43  Me=3,7 | 2,93±3,44  Me=1,9 | 6,22±5,33  Me=5,75 |
| 3 | 4,72±9,33  Me=3,85 | 13,67±7,1  Me=13,1 | 9,92±3,66  Me=11,1 |
| 4 | 3,38±6,99  Me=3,85 | 18,07±5,8  Me=16,65 | 11,9±4,55  Me=10,7 |
| 5 | 4,68±6,72  Me=3,55 | 19,37±6,45  Me=16,95 | 14,42±5,75  Me=12,9 |
| 6 | 6,95±3,13  Me=7,4 | 24,47±10  Me=23,75 | 15,67±3,91  Me=14,85 |
| 7 | 10,73±5,59  Me=7,4 | 38,92±9,99  Me=38,9 | 19,52±5,5  Me=19,25 |
| 8 | 11,93±5,43  Me=11 | 32,95±8,96  Me=32,05 | 20,75±5,65  Me=20,5 |
| 9 | 10,78±6,88  Me=11,1 | 40,18±9,23  Me=-37,75 | 18,88±4,64  Me=19,25 |
| 10 | 10,78±6,09  Me=9,3 | 37,68±11,02  Me=-34,5 | 18,78±4,17  Me=18,4 |
| 11 | 3,32±7,38  Me=2 | 5,6±7,7  Me=1,85 | 6,3±3,94  Me=7,4 |
| 12 | 1,05±4,12  Me=0 | -1,62±3,08  Me=0 | -0,53±4,95  Me=0 |

**Table E. Descriptive statistics concerning the effect of long-term administration of magnesium lactate in micronized form (micro) or normal form on the analgesic activity of tramadol (TRAM)**

| **Compound** | **30’** | **45’** | **60’** | **120’** |
| --- | --- | --- | --- | --- |
| Magnesium lactate normal 7,5 | 1,31±0,12  Me=1,31 | 1,64±0,21  Me=1,69 | 2,26±0,47  Me=2,29 | 4,93±0,39  Me=4,98 |
| Magnesium lactate micro 7,5 | 1,72±0,23  Me=1,74 | 2,33±0,39  Me=2,43 | 3,06±0,44  Me=3,06 | 5,95±0,39  Me=5,98 |
| Magnesium lactate normal 15 | 1,39±0,16  Me=1,36 | 1,92±0,46  Me=1,81 | 3,36±0,47  Me=3,33 | 7,26±0,15  Me=7,26 |
| Magnesium lactate micro 15 | 2,23±0,43  Me=2,33 | 2,75±0,57  Me=2,74 | 4,41±0,26  Me=4,5 | 8,58±0,43  Me=8,42 |
| Control | 0,49±0,14  Me=0,47 | 0,62±0,21  Me=0,63 | 0,86±0,14  Me=0,88 | 0,99±0,11  Me=0,98 |

**Table F. Descriptive statistics concerning the effect of magnesium lactate in normal and micronized forms at doses of 7.5 and 15 mg**

**kg^-1^ of magnesium ions kg^-1^ body mass on the concentration of ionized fraction of magnesium**
